# Annexin A6 Modulates Chick Cranial Neural Crest Cell Emigration

**DOI:** 10.1371/journal.pone.0044903

**Published:** 2012-09-11

**Authors:** Chyong-Yi Wu, Lisa A. Taneyhill

**Affiliations:** Department of Animal and Avian Sciences, University of Maryland, College Park, Maryland, United States of America; Seattle Children's Research Institute, United States of America

## Abstract

The vertebrate neural crest is a population of migratory cells that originates in the dorsal aspect of the embryonic neural tube. These cells undergo an epithelial-to-mesencyhmal transition (EMT), delaminate from the neural tube and migrate extensively to generate an array of differentiated cell types. Elucidating the gene regulatory networks involved in neural crest cell induction, migration and differentiation are thus crucial to understanding vertebrate development. To this end, we have identified Annexin A6 as an important regulator of chick midbrain neural crest cell emigration. Annexin proteins comprise a family of calcium-dependent, membrane-binding molecules that mediate a variety of cellular and physiological processes including cell adhesion, migration and invasion. Our data indicate that Annexin A6 is expressed in the proper spatio-temporal pattern in the chick midbrain to play a potential role in neural crest cell ontogeny. To investigate Annexin A6 function, we have depleted or overexpressed Annexin A6 in the developing midbrain neural crest cell population. Our results show that knock-down or overexpression of Annexin A6 reduces or expands the migratory neural crest cell domain, respectively. Importantly, this phenotype is not due to any change in cell proliferation or cell death but can be correlated with changes in the size of the premigratory neural crest cell population and with markers associated with EMT. Taken together, our data indicate that Annexin A6 plays a pivotal role in modulating the formation of cranial migratory neural crest cells during vertebrate development.

## Introduction

Neural crest cells are a population of migratory cells in the developing vertebrate embryo. In the chick embryo, these cells initially reside in the most dorsal region of the neural tube as premigratory neural crest cells that subsequently undergo an epithelial-to-mesencyhmal transition (EMT) to become motile. These migratory cells then traverse stereotypical pathways in both the head and trunk and later differentiate to form a wide variety of structures in the embryo, including the craniofacial skeleton, components of the peripheral nervous system and heart, and skin pigment cells [1]. Because of the contributions of neural crest cells to multiple derivatives, it is critical to study how these cells arise in the developing embryo, including the role of various genes in controlling the induction, migration, and differentiation of the neural crest.

To this end, we explored a potential role for Annexin A6 in neural crest cell development and find that Annexin A6 functions in controlling neural crest cell emigration in the developing chick midbrain. *Annexins* are a large multi-gene family (more than 160 family members) whose protein products bind to calcium and phospholipids in a reversible manner in order to mediate diverse cellular processes, including vesicle trafficking, calcium signaling, cell migration, and cell proliferation [2], [3]. Each annexin contains an N-terminal interaction domain for association with other proteins that is subject to post-translational modifications [4]. The membrane binding domain of annexins is referred to as the annexin core, which contains four repeats of a conserved 70 amino acid sequence and in turn associates peripherally with the plasma membrane through the recruitment of calcium ions [4]. Annexin A6 possesses two of these cores, allowing the protein to bind to one or two membranes [5], [6]. Annexin A6 was first identified in the matrix vesicles of chicken growth plate cartilage [7], and recent research has documented Annexin A6 expression in a wide range of mammalian tissues, including skeletal muscle, heart, and spleen (for review, see [3]) and in some cancer cell lines [8], [9]. As such, Annexin A6 has diverse functions depending upon the tissue context, including endosomal transport [10], caveolae formation [11], [12], reorganization of the actin cytoskeleton [13], [14], down-regulation of the EGFR/MAPK pathway [9], [15], [16], and regulation of cell adhesion, migration and invasiveness [17].

Here we report the first characterization of Annexin A6 in the chick embryo with respect to its expression profile and function during neural crest ontogeny in the midbrain. Through whole-mount *in situ* hybridization, we find that *Annexin A6* transcripts are localized to the chick neural tube, ectoderm, and in migratory neural crest cells. Importantly, knock-down or overexpression of Annexin A6 attenuates or enhances neural crest cell emigration, respectively. Importantly, this effect on the migratory neural crest cell domain can be correlated with concomitant changes in the size of the premigratory neural crest cell population and with molecular markers associated with EMT. Collectively, our studies reveal a novel function for an annexin family member in controlling midbrain neural crest cell emigration in the developing chick embryo.

## Results

### *Annexin A6* Transcripts are Present in Premigratory and Migratory Neural Crest Cells

**Figure 1:**
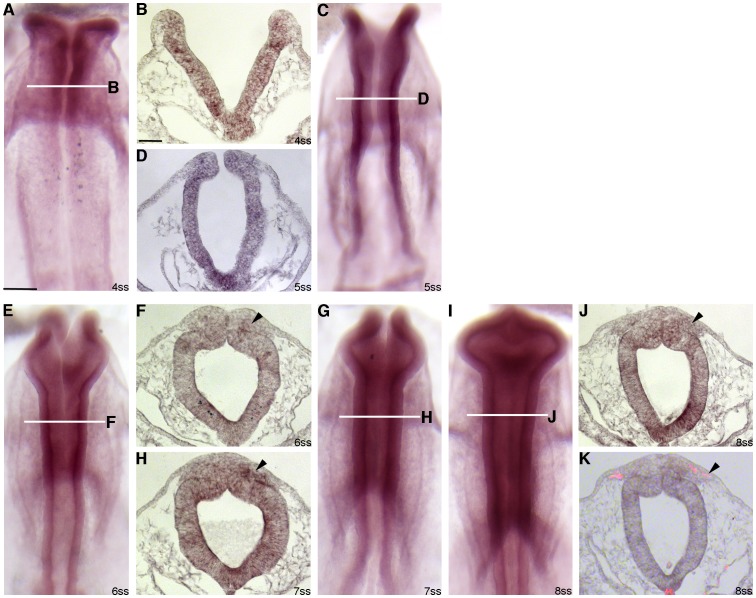
*Annexin A6* transcript distribution in the developing chick embryo. (A,C,E,G,I) Whole-mount *in situ* hybridization for *Annexin A6* followed by indicated transverse sections (B,D,F,H,J) shown for specific developmental stages (A,B: 4 ss; C,D: 5 ss; E,F: 6 ss; G,H: 7 ss; I–J: 8 ss). (K) Representative midbrain transverse section taken from an 8 ss embryo that has undergone whole-mount *in situ* hybridization for *Annexin A6* followed by sectioning and immunohistochemistry for the migratory neural crest cell marker HNK-1 (red). Arrowheads in (F,H,J,K) indicate *Annexin A6* mRNA in migratory neural crest cells. Scale bars in (A) and (B) are 200 and 50 µm, respectively, and are applicable to respective image types.

In order to ascertain a potential role for Annexin A6 in chick midbrain neural crest cell development, we performed whole-mount *in situ* hybridization, in conjunction with transverse sectioning, to document the expression profile of *Annexin A6* in the developing embryo. *Annexin A6* was observed as early as the 4 somite stage (ss), with transcripts localized to the dorsal neural folds/premigratory neural crest (Fig. 1) and trunk (data not shown), with lower levels seen in the head ectoderm. In transverse sections through the midbrain region of embryos, we noted *Annexin A6* expression throughout the neural tube (Fig. 1B,D,F,H,J,K) and in newly migratory neural crest cells (Fig. 1F,H,J,K; arrowheads), a finding corroborated by immunostaining with the migratory neural crest cell marker HNK-1 at the 8 ss (Fig. 1K; arrowhead). Collectively, our results indicate that *Annexin A6* is expressed in the proper spatio-temporal pattern to play a role in neural crest cell development.

### Knock-down of Annexin A6 reduces the size of the premigratory neural crest cell domain and abrogates neural crest cell EMT, thereby negatively impacting neural crest cell migration *in vivo*

**Figure 2:**
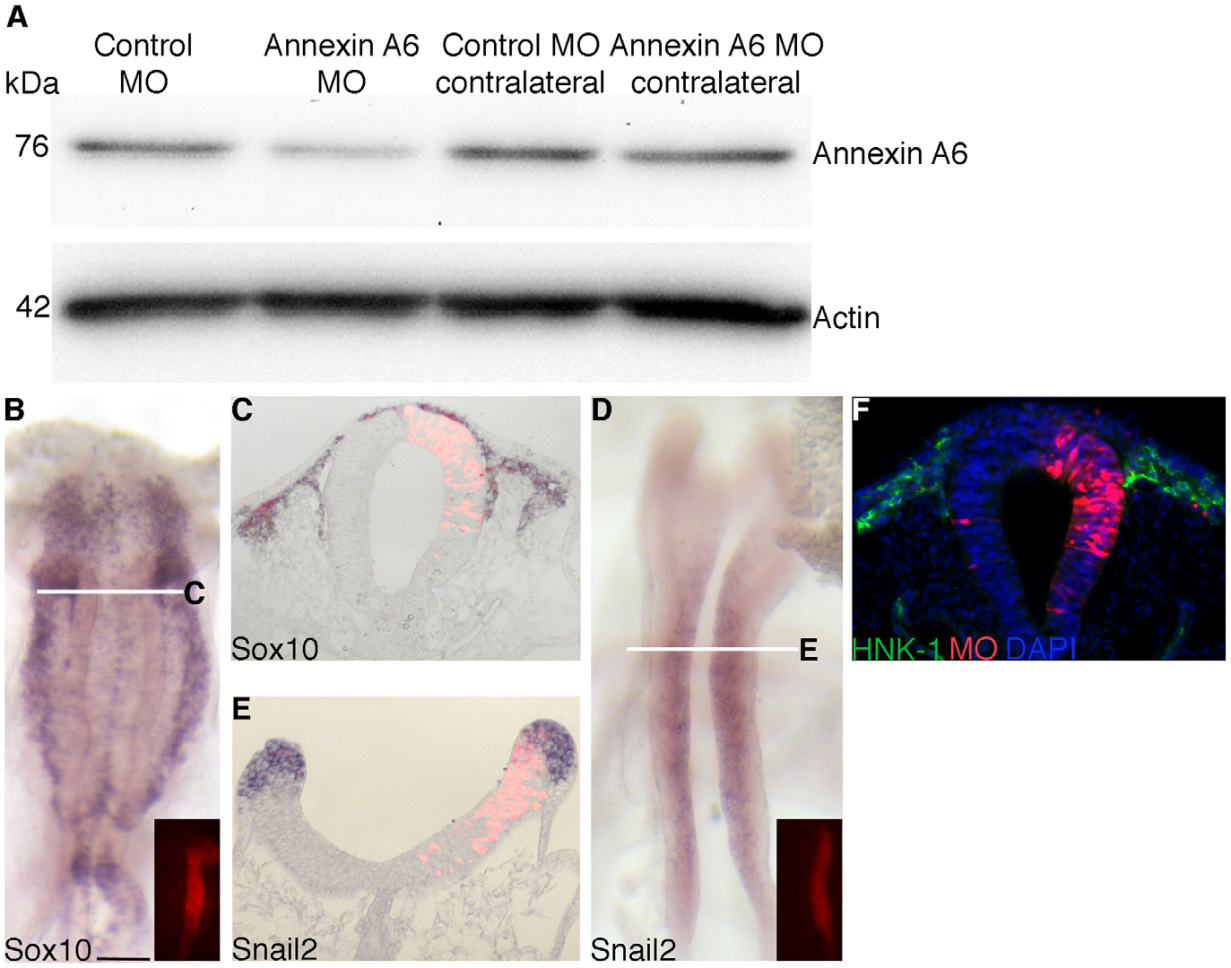
A 5 base pair mismatch *Annexin A6* control MO does not affect chick midbrain neural crest cell emigration *in vivo*. (A) Immunoblots for Annexin A6 and β-actin showing reduced levels of Annexin A6 protein upon *Annexin A6* MO treatment, with no change observed upon control MO treatment. (B,D) Whole-mount *in situ* hybridization followed by indicated transverse sections for *Sox10* (C) and *Snail2* (E), respectively, after 8 hour incubation following treatment with control MO. (F) Representative transverse section taken from an embryo treated with control MO for 8 hours followed by immunohistochemistry for HNK-1 (green). No change is noted in whole-mount images or sections with respect to neural crest cell emigration or migration. In all experiments, the right side of the embryo is electroporated, as indicated by the lissamine (red) fluorescence of the MO in the transverse sections (C,E) and/or in the inset images of each whole-mount (B,D). Scale bar in (B) is 50 µm and applicable to all whole-mount and section images. MO, red; DAPI, blue.

To elucidate a functional role for Annexin A6 in the cranial neural crest, we depleted Annexin A6 from the developing neural crest cell population of the chick midbrain using a morpholino antisense oligonucleotide (MO) designed to target the sequence surrounding the *Annexin A6* translational start site. Using the method of *in ovo* electroporation [18], [19], [20] we electroporated chick midbrain neural tube cells with a 5 base pair (bp) mismatch *Annexin A6* control MO (hereafter referred to as control MO). The control MO had no effect on Annexin A6 protein levels in the neural tube (Fig. 2A). Electroporation of *Annexin A6* MO, however, depleted Annexin A6 protein in the neural tube by approximately 2-fold (Fig. 2A), a result in good agreement with what we have observed previously [21].

**Figure 3:**
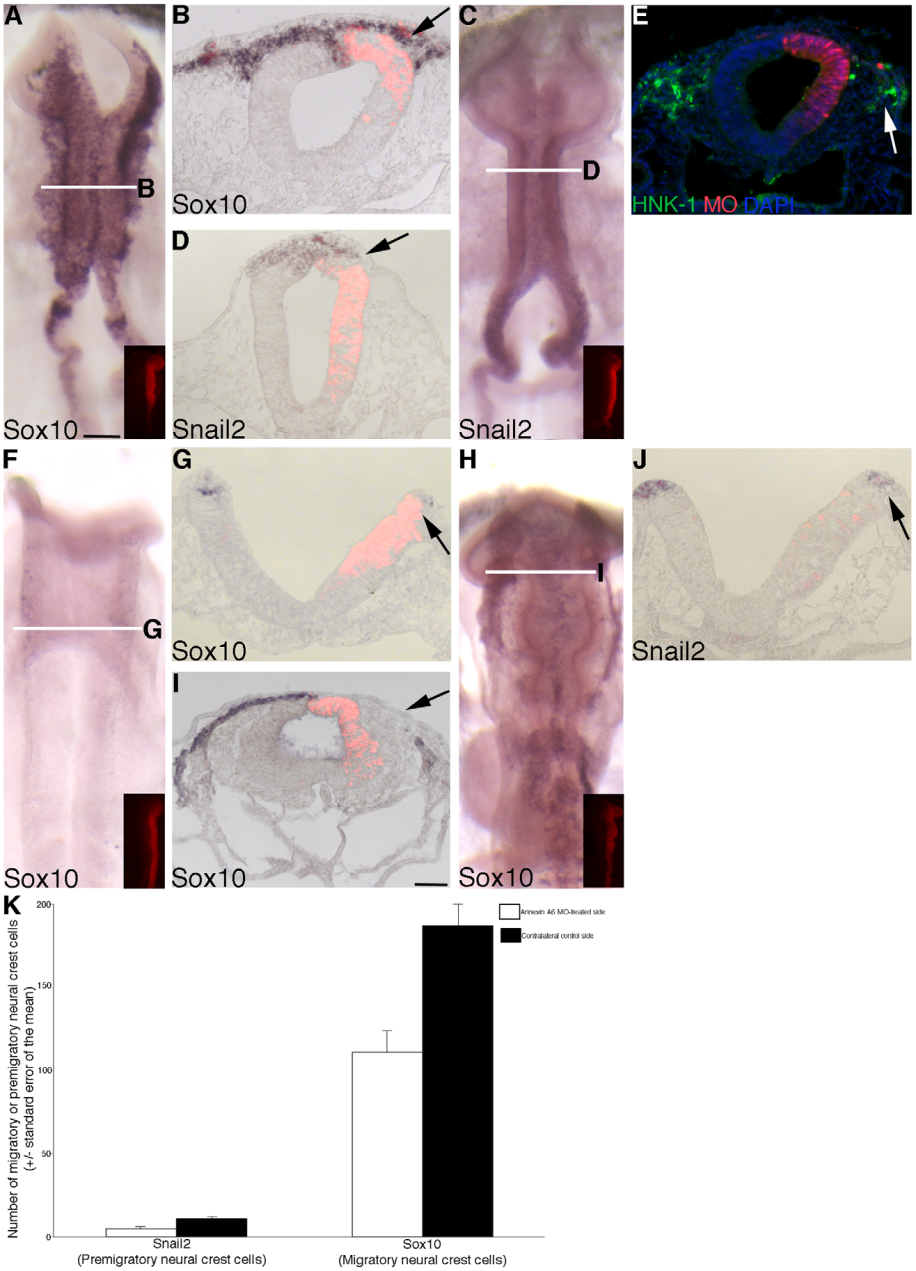
MO-mediated depletion of Annexin A6 from the developing neural crest cell population of the chick midbrain diminishes the size of the premigratory and migratory neural crest cell domains. (A,C) Whole-mount *in situ* hybridization followed by indicated transverse sections for *Sox10* (B) and *Snail2* (D), respectively, after 8 hour incubation following treatment with *Annexin A6* MO. (E) Representative transverse section taken from an embryo treated with *Annexin A6* MO for 8 hours followed by immunohistochemistry for HNK-1 (green). (F,H) Whole-mount *in situ* hybridization followed by indicated transverse section (G,I) for *Sox10* after 3 and 20 hour incubation following treatment with *Annexin A6* MO, respectively. (J) Representative transverse section taken from an embryo treated with *Annexin A6* MO for 3.5 hours followed by *Snail2* whole-mount *in situ* hybridization. Arrows in (B–J) indicate the migratory or premigratory neural crest cell domain. In all experiments, the right side of the embryo is electroporated, as indicated by the lissamine (red) fluorescence of the MO in the transverse sections (B,D,E,G,I,J) and/or in the inset images of each whole-mount (A,C,F,H). (K) Graphical representation of changes in the premigratory (*Snail2*) and migratory (*Sox10*) neural crest cell populations upon depletion of Annexin A6. Scale bar in (A) is 50 µm and applicable to all whole-mount and section images except for that shown in (I) where the scale bar is also 50 µm. MO, red; DAPI, blue.

To examine any potential differences in the migratory neural crest cell population, embryos were electroporated with either *Annexin A6* or control MO, re-incubated for 8 hours, and then processed for whole-mount *in situ* hybridization for *Sox10* and *Snail2*, or immunostained using an antibody to HNK-1. Treatment with control MO has no effect on *Sox10* (Fig. 2B,C; 12/12 embryos), *Snail2* (Fig. 2D,E; 6/6 embryos) and HNK-1 immunostaining (Fig. 2F; 9/9 embryos). Depletion of Annexin A6 from the premigratory neural crest, however, negatively affected neural crest cell migration, as determined by a reduction in the *Sox10*-positive migratory neural crest domain on the electroporated side (right) of the embryo, compared to the contralateral control side (and to control embryos) (Fig. 3A,B, arrows; 8/11 embryos). We also assessed the expression of *Snail2*, a molecular marker of premigratory and migratory cranial neural crest cells, and we observed a decrease in the *Snail2*-positive migratory neural crest cell domain on the electroporated side (Fig. 3C,D, arrows; 8/12 embryos) compared to the contralateral control side (and to control embryos). This same observation is made for the migratory neural crest cell marker HNK-1 (Fig. 3E, arrow; 10/13 embryos). Short-term (3 hours; Fig. 3F,G, arrow, 8/8 embryos) and long-term (20 hours; Fig. 3H,I, arrow, 6/6 embryos) depletion of Annexin A6 also reduced the *Sox10*-positive migratory neural crest cell domain. No differences were apparent at either of these time points upon treatment with the control MO (7/7 and 6/6 embryos, respectively, data not shown). Taken together, these data suggest that reduced Annexin A6 levels diminish neural crest cell migration in the chick midbrain.

**Figure 4:**
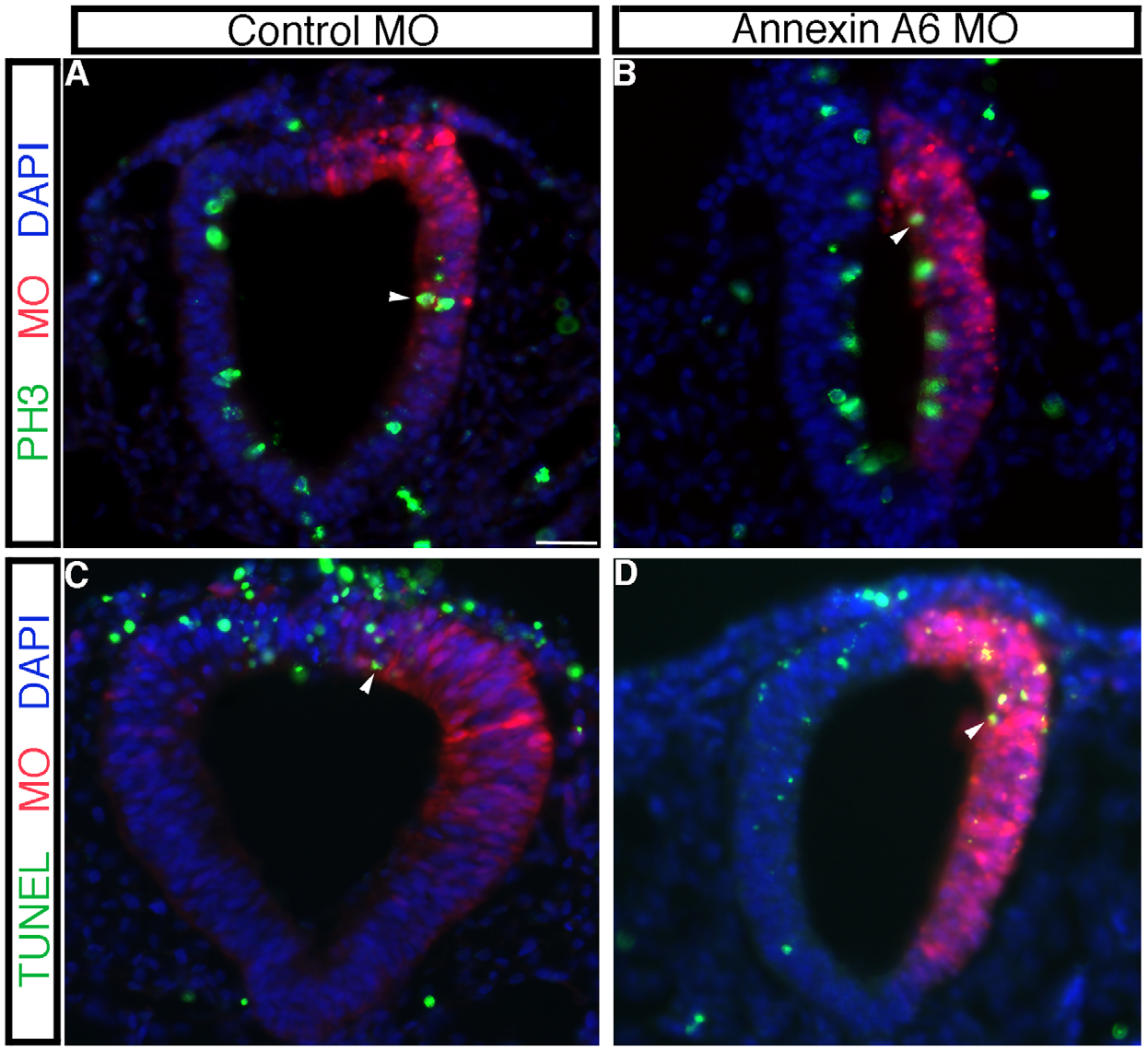
Depletion of Annexin A6 does not alter cell proliferation or cell death in the chick embryonic neural tube or migratory neural crest cell population. (A–D) Electroporation of control (A,C) or *Annexin A6* (B,D) MO, followed by 8 hour incubation, transverse sectioning, and processing for phospho-histone H3 immunohistochemistry (A,B, PH3, green) or TUNEL (C,D, green) (representative sections are shown). Arrowheads indicate PH3-positive (A,B) or TUNEL-positive (C,D) nuclei, with a similar distribution observed in the neural tube and in migratory neural crest cells in the presence of either MO and with that found on the contralateral control side of the embryo. In all experiments, the right side of the embryo is electroporated with the MO, as indicated by the lissamine (red) fluorescence of the MO in the sections. Scale bar in (A) is 50 µm and applicable to all images. MO, red; DAPI, blue.

To determine the mechanism by which Annexin A6 knock-down was affecting neural crest cell emigration and migration, we examined treated embryos for changes in the premigratory neural crest cell population, cell proliferation and cell death. We noted a reduction in the premigratory neural crest cell population in embryos treated with *Annexin A6* MO upon examination of *Snail2* expression in the dorsal neural tube prior to neural tube closure (and thus before neural crest cell emigration/migration) (Fig. 3J, arrow; 8/10 embryos) compared to those treated with the control MO (13/13 embryos, data not shown). To quantify our observations, we calculated the number of *Snail2*- or *Sox10*-positive cells in the premigratory or migratory neural crest domain, respectively, on both the electroporated and contralateral control sides in at least 7 serial sections obtained from a minimum of 3 embryos treated with *Annexin A6* or control MO, as carried out previously in [19], [20], [22]. Our data indicated that depletion of Annexin A6 results in a statistically significant 2.2- and 1.7-fold decrease, respectively, in the *Snail2-* and *Sox10-*positive premigratory and migratory neural crest cell domains compared to the contralateral control side (Fig. 3K; *Snail2*-positive cells: control side  = 11+/−1, *Annexin A6* MO side  = 5+/−1; *Sox10*-positive cells: control side  = 187+/−14, *Annexin A6* MO side  = 111+/−8), with a Student’s t test of *p*<0.000001 for *Snail2* and *p*<0.0001 for *Sox10*. No statistically significant difference was observed in the presence of control MO compared to the contralateral control side (*Snail2*-positive cells: control side  = 30+/−4, control MO side  = 29+/−4, fold difference of 0.98; *Sox10*-positive cells: control side  = 114+/−8; control MO side  = 115+/−7, fold difference of 1.0). We next performed phospho-histone H3 (PH3) immunostaining on transverse sections taken from embryos treated with *Annexin A6* or control MO for 8 hours. No difference in cell proliferation was observed in the presence of either MO, compared to each other and the contralateral control side of the embryo (Fig. 4A,B, arrowheads; 6/6 embryos for each treatment). In addition, cell death was not altered upon treatment with either MO based upon TUNEL assay and analysis (Fig. 4C,D, arrowheads; 7/7 and 6/6 embryos, respectively, for *Annexin A6* and control MO treatments). Furthermore, examination of the *Sox10* and *Snail2 in situ* hybridization brightfield images and immunohistochemical images for HNK-1 at a higher magnification revealed no changes in neural crest cell size and/or architecture (data not shown).

**Figure 5:**
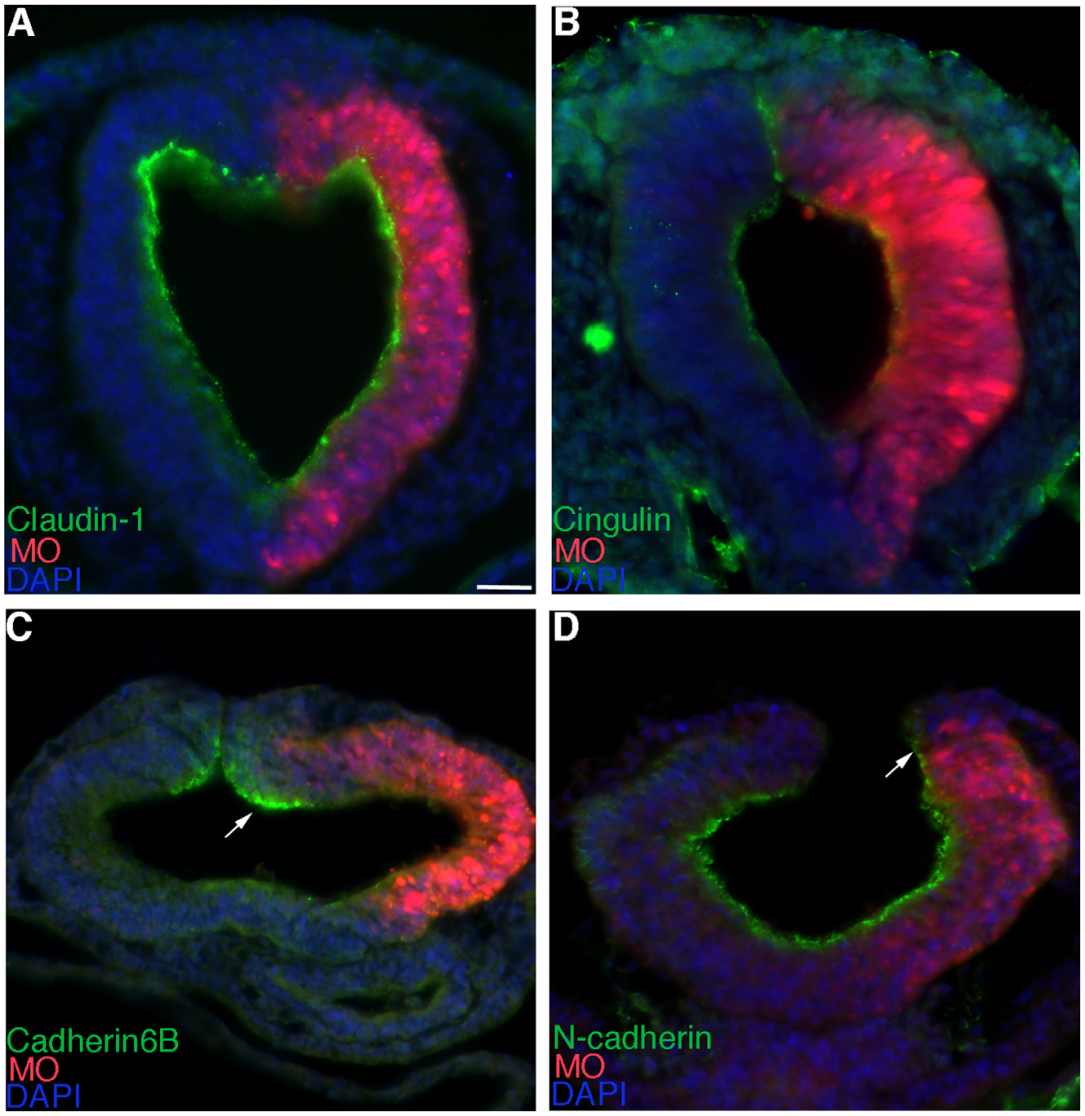
MO-mediated depletion of Annexin A6 leads to retention of molecular markers of adherens junctions. (A–D) Representative transverse section taken through the midbrain of an embryo electroporated with *Annexin A6* MO (red) after 6 (A–C) and 5 (D) hours of incubation and processing by immunohistochemistry for Claudin-1 (A), Cingulin (B), Cadherin6B (C), and N-cadherin (D) (all green), respectively. Arrows denote the maintenance of proteins on the electroporated side (right) of the neural tube. Scale bar in (A) is 50 µm and applicable to all images. MO, red; DAPI, blue.

In order to investigate whether Annexin A6 knock-down reduced neural crest cell emigration by altering the process of EMT, we examined the distribution of several adherens and tight junction proteins whose down-regulation has been previously correlated with neural crest cell EMT/migration [19,20,21,22, our unpublished data] (Fig. 5). We find that depletion of Annexin A6 does not affect the distribution of the tight junction proteins Claudin-1 or Cingulin (Fig. 5A,B; 4/4 embryos for each), but instead results in the retention of the adherens junction proteins Cadherin6B and N-cadherin (Fig. 5C,D, arrows; Cadherin6B, 5/6 embryos; N-cadherin, 8/10 embryos), with no differences observed in control MO-treated embryos (data not shown). These results indicate that Annexin A6 knock-down, directly or indirectly, disrupts the EMT program at the level of the adherens junctions and point to a potential role for Annexin A6 in controlling neural crest cell EMT and emigration. Collectively, our results suggest that Annexin A6 modulates neural crest cell emigration, and subsequent migration, and that this function may be related to a reduction in the size of the premigratory neural crest cell domain as well as direct or indirect effects on the molecular program associated with neural crest cell EMT.

### Overexpression of Annexin A6 increases the size of the premigratory neural crest cell domain and enhances neural crest cell EMT, leading to an expansion of neural crest cell migration *in vivo*</p>

**Figure 6:**
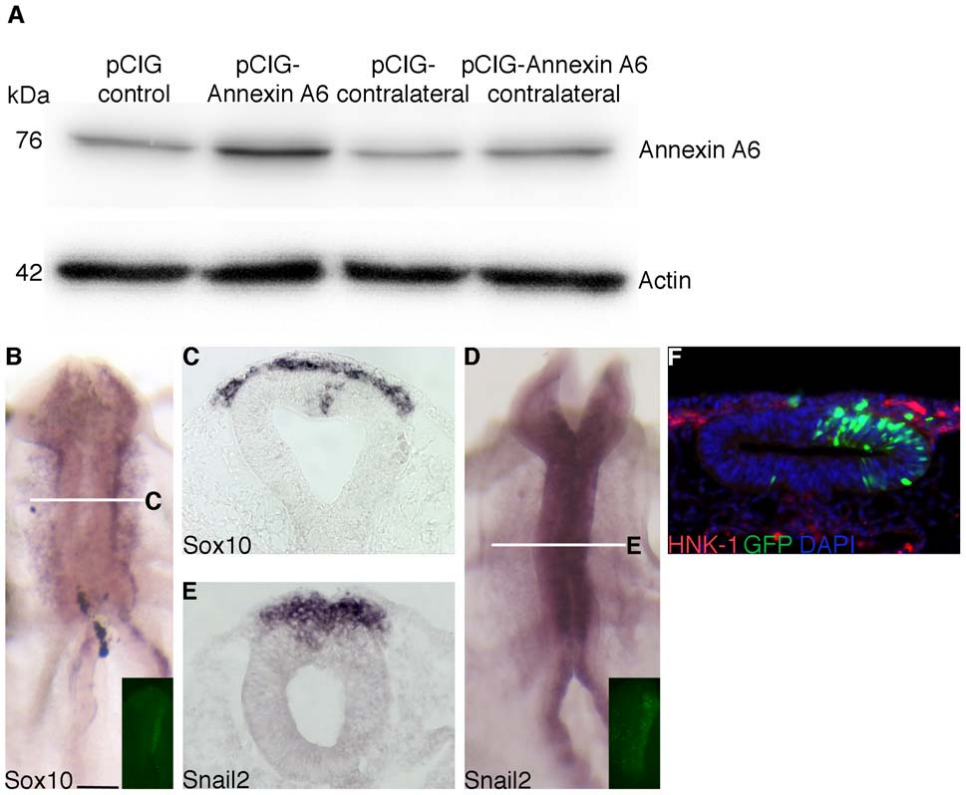
The pCIG control expression construct does not affect chick midbrain neural crest cell emigration *in vivo*. (A) Immunoblots for Annexin A6 and β-actin showing increased levels of Annexin A6 protein upon pCIG-Annexin A6 treatment, with no change observed upon pCIG treatment. (B,D) Whole-mount *in situ* hybridization followed by indicated transverse sections (C,E) for *Sox10* and *Snail2*, respectively, after 8 hour treatment with pCIG. (F) Representative transverse section taken from an embryo treated with pCIG for 8 hours followed by whole-mount immunohistochemistry for HNK-1 (red). No change is noted in whole-mount images or sections with respect to neural crest cell emigration or migration. In all experiments, the right side of the embryo is electroporated with pCIG, as indicated by the GFP (green) fluorescence in the transverse section (F) and/or in inset images of each whole-mount (B,D). Scale bar in (B) is 50 µm and applicable to all images. GFP, green; DAPI, blue.

We cloned the full-length chick *Annexin A6* cDNA into a chick expression construct (pCIG) and introduced the construct into the premigratory neural crest cell population of the chick midbrain by electroporation. Overexpression of AnnexinA6 in the neural tube leads to an approximate 2-fold increase in the amount of Annexin A6 protein (Fig. 6A), as observed previously [21], with no change observed upon treatment with the pCIG construct.

**Figure 7:**
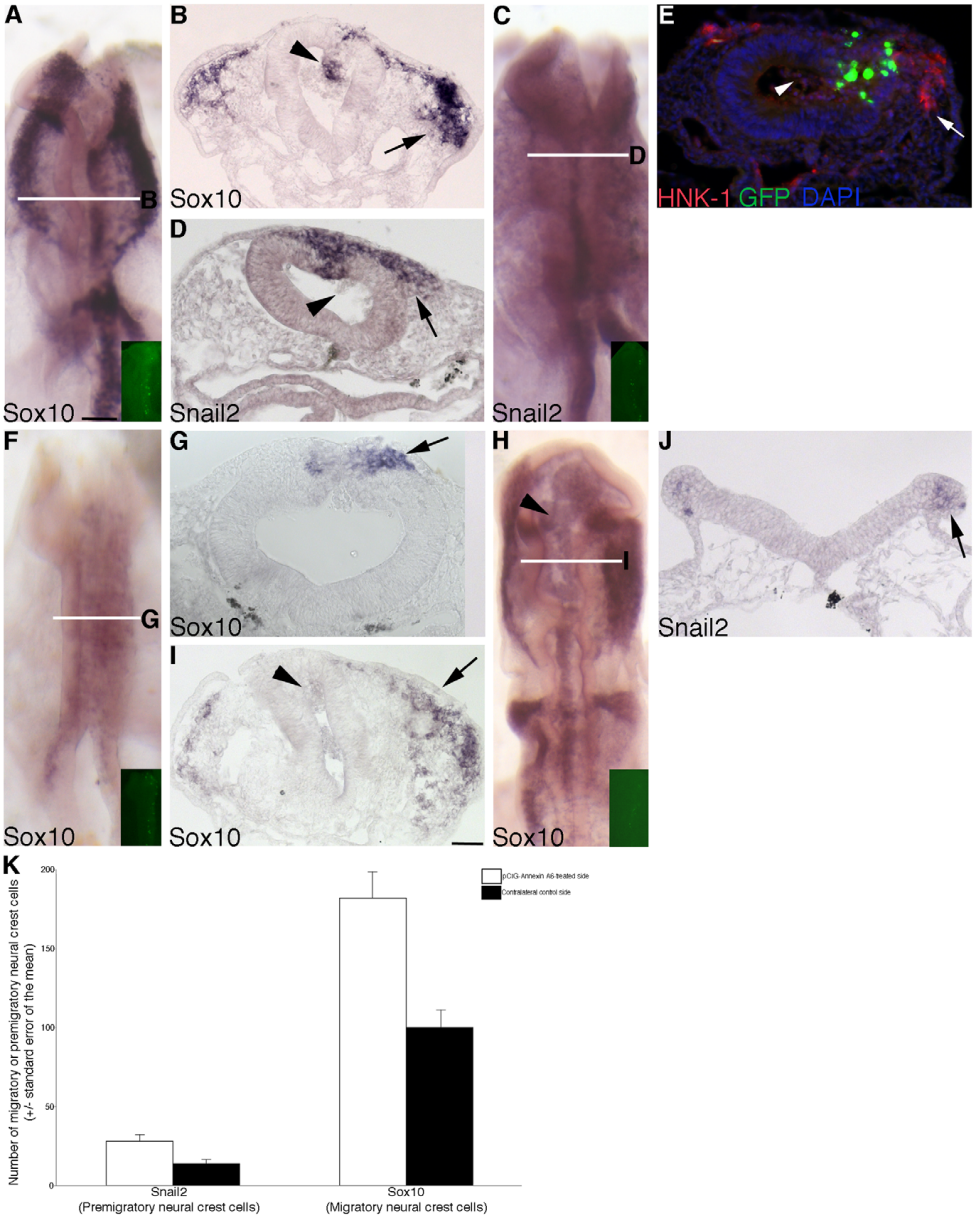
Overexpression of Annexin A6 in the developing neural crest cell population of the chick midbrain increases the size of the premigratory and migratory neural crest cell domains. (A,C) Whole-mount *in situ* hybridization followed by indicated transverse sections for *Sox10* (B) and *Snail2* (D), respectively, after 8 hour incubation following treatment with pCIG-Annexin A6. (E) Representative transverse section taken from an embryo treated with pCIG-Annexin A6 for 8 hours followed by immunohistochemistry for HNK-1 (red). (F,H) Whole-mount *in situ* hybridization followed by indicated transverse section (G,I) for *Sox10* after 4 and 20 hour incubation following treatment with pCIG-Annexin A6, respectively. (J) Representative transverse section taken from an embryo treated with pCIG-Annexin A6 for 6 hours followed by *Snail2* whole-mount *in situ* hybridization. Arrows in (B–J) indicate the migratory or premigratory neural crest cell domain. In all experiments, the right side of the embryo is electroporated, as indicated by the GFP (green) fluorescence of the expression construct in (E) and/or in the inset images of each whole-mount (A,C,F,H). (K) Graphical representation of changes in the premigratory (*Snail2*) and migratory (*Sox10*) neural crest cell populations upon Annexin A6 overexpression. Scale bar in (A) is 50 µm and applicable to all whole-mount and section images except for that shown in (I) where the scale bar is also 50 µm. GFP, green; DAPI, blue.

To assess whether neural crest cell migration was affected upon overexpressing Annexin A6, we electroporated embryos with either the control pCIG or pCIG-Annexin A6 construct, re-incubated them for 8 hours, and then processed embryos as described previously for the MO electroporations. Treatment with the pCIG control construct had no effect on *Sox10* (Fig. 6B,C; 13/13 embryos), *Snail2* (Fig. 6D,E; 7/7 embryos), and HNK-1 staining (Fig. 6F; 8/8 embryos). Overexpression of Annexin A6, however, enhanced neural crest cell migration, as evidenced by the increase in the *Sox10*- (13/14 embryos) and *Snail2*-positive (15/17 embryos) migratory neural crest cell domains on the electroporated side (right), compared to the contralateral control side (and to control embryos) (Fig. 7A,B for *Sox10*, Fig. 7C,D for *Snail2*; arrows). This phenotype is also evident upon immunostaining Annexin A6-overexpressing embryos with HNK-1 (Fig. 7E, arrow; 10/13 embryos). In addition, we often observed cells within the neural tube lumen that were either positive (*Sox10*, Fig. 7B,E,I, arrowheads) or negative (*Snail2*, Fig. 7D) for neural crest molecular markers. Finally, at earlier (4 hours; Fig. 7F,G; 7/9 embryos) and later (20 hours; Fig. 7H,I; 9/9 embryos) time points post-electroporation, we noted precocious emigration of neural crest cells (Fig. 7G, arrow) and an increase in the *Sox10*-positive migratory neural crest cell domain (Fig. 7G,I, arrows), respectively, which are phenotypes not observed with the pCIG controls (4 hours, 7/8 embryos; 20 hours, 8/8 embryos**,** data not shown). All together, these data indicate a role for Annexin A6 in augmenting neural crest cell emigration and migration.

**Figure 8:**
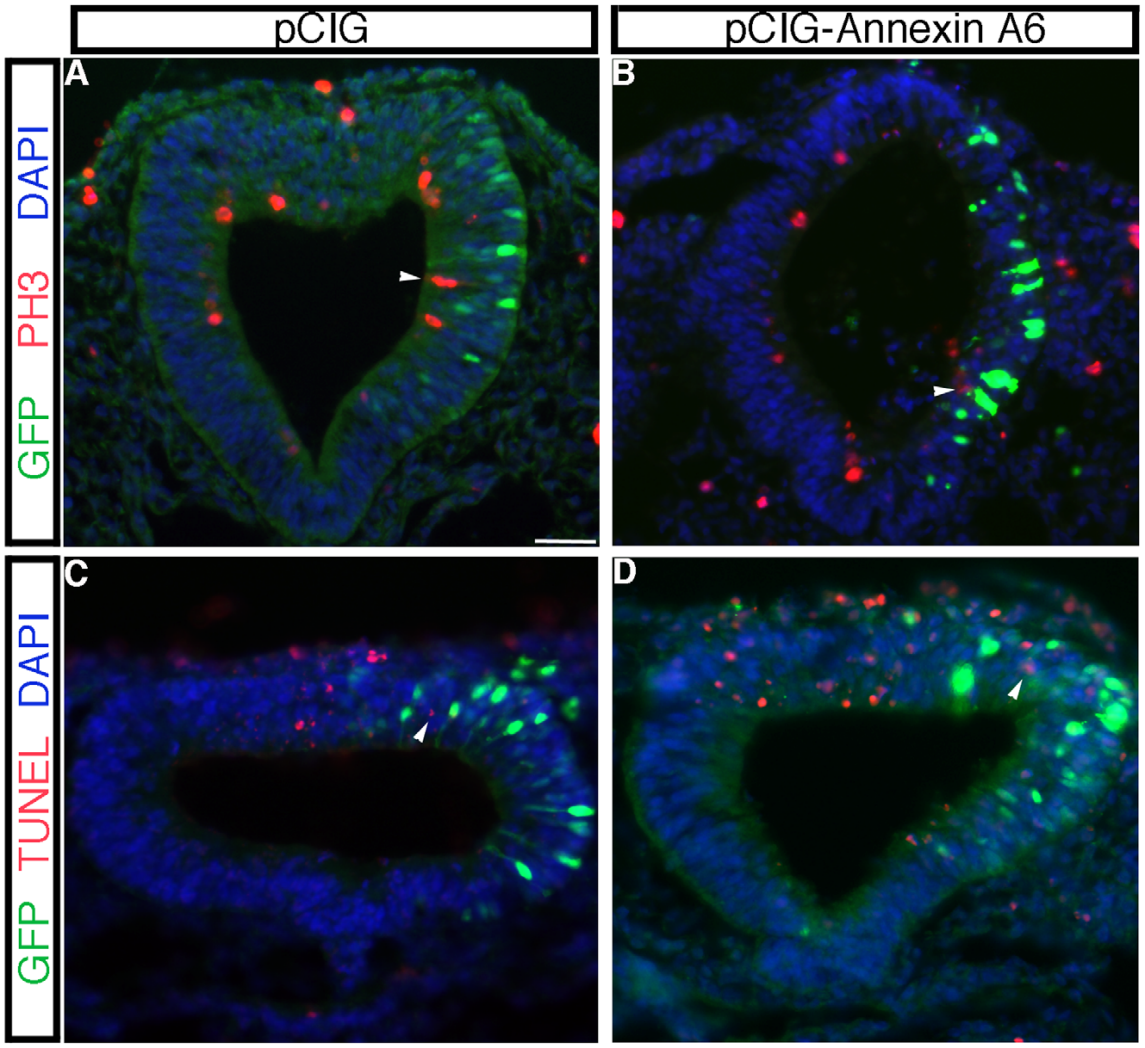
Annexin A6 overexpression does not alter cell death nor cell proliferation in the chick embryonic neural tube or migratory neural crest cell population. (A–D) Electroporation of the pCIG control (A,C) or pCIG-Annexin A6 (B,D) constructs, followed by 8 hour incubation, transverse sectioning, and processing for phospho-histone H3 immunohistochemistry (A,B, PH3, red) or TUNEL (C,D, red) (representative sections are shown). Arrowheads indicate PH3-positive (A,B) or TUNEL-positive (C,D) nuclei, with a similar distribution in the neural tube and in migratory neural crest cells observed in the presence of either construct and with that found on the contralateral control side of the embryo. In all experiments, the right side of the embryo is electroporated with the construct, as indicated by the GFP (green) fluorescence in the sections. Scale bar in (A) is 50 µm and applicable to all images. GFP, green; DAPI, blue.

To determine how Annexin A6 overexpression modulates neural crest cell emigration and migration, we examined embryos for changes in the premigratory neural crest cell domain, cell proliferation and cell death. Overexpression of Annexin A6 caused an expansion of the premigratory neural crest cell population, as assessed by *Snail2* expression in young embryos prior to neural fold fusion (Fig. 7J, arrow; 11/12 embryos), compared to those treated with the control pCIG construct (7/8 embryos, data not shown). In order to quantify effects on neural crest cell emigration and migration, we calculated the number of *Snail2*- or *Sox10*-positive cells in the premigratory or migratory neural crest cell domains, respectively, on both the electroporated and contralateral control sides in at least 7 serial sections obtained from a minimum of 3 embryos treated with pCIG or pCIG-Annexin A6 (Fig. 7K). Our data revealed that overexpression of Annexin A6 results in a statistically significant 2.1- and 1.8-fold increase in the *Snail2*- and *Sox10*-positive premigratory and migratory neural crest cell domains, respectively, compared to the contralateral control side (*Snail2*-positive cells: control side  = 14+/−2, pCIG-Annexin A6 side  = 28+/−3; *Sox10*-positive cells: control side  = 100+/−9, pCIG-Annexin A6 = 182+/−15), with a Student’s t test of *p*<0.0001 for both *Snail2* and *Sox10*. No statistically significant difference was observed in the presence of pCIG compared to the contralateral control side (*Snail2*-positive cells: control side  = 65+/−4, pCIG control side  = 65+/−5, fold difference of 1.0; *Sox10*-positive cells: control side  = 209+/−16; pCIG control side  = 211+/−17, fold difference of 1.0). These data are in good agreement with those obtained in our MO knock-down experiments. We then performed PH3 immunostaining and a TUNEL assay and noted no change in cell proliferation (Fig. 8A,B, arrowheads; 10/11 embryos) or cell death (Fig. 8C,D, arrowheads; 4/5 embryos). Finally, there were no apparent alterations in the size and/or architecture of migratory neural crest cells (data not shown).

**Figure 9:**
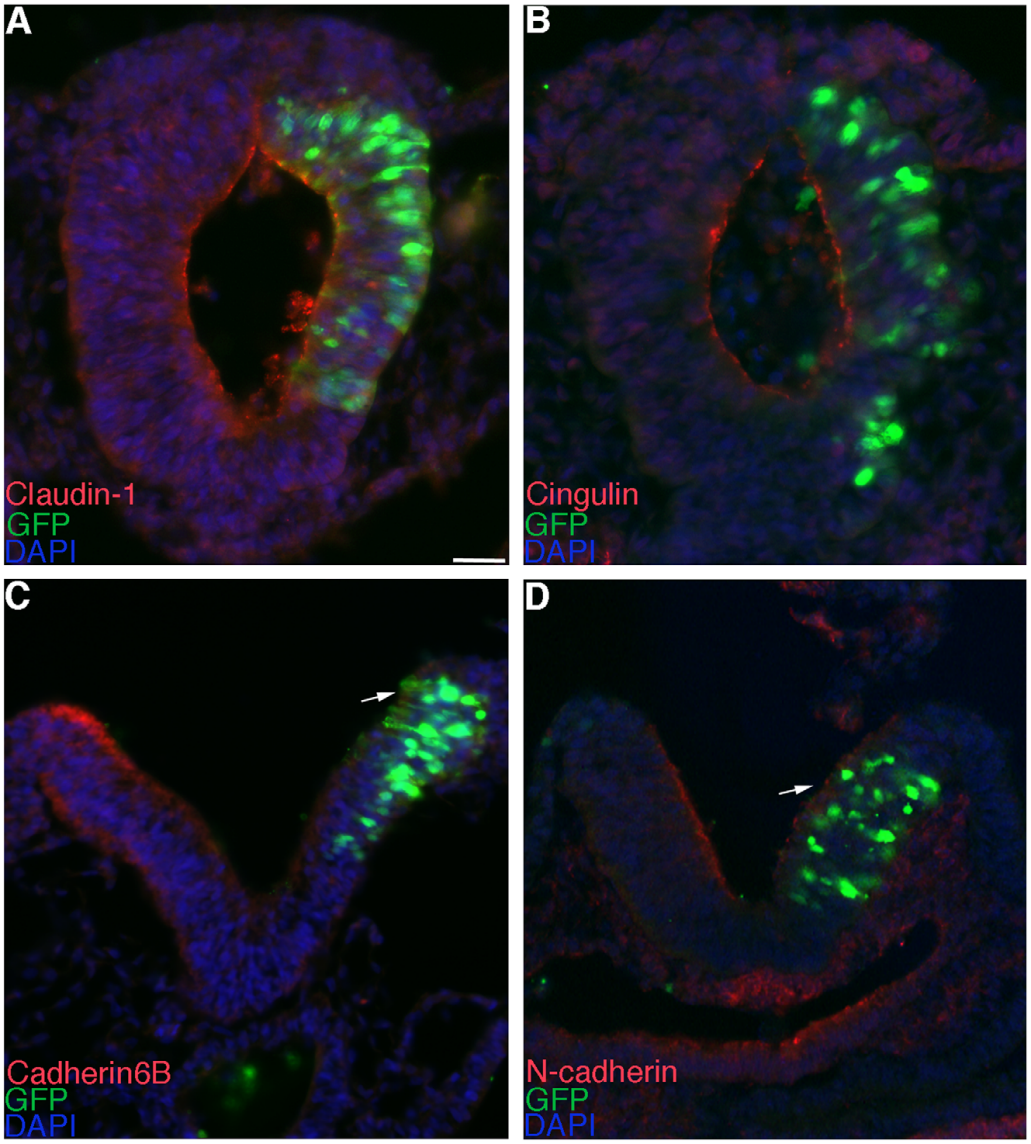
Annexin A6 overexpression leads to precocious loss of molecular markers of adherens junctions. (A–D) Representative transverse section taken through the midbrain of an embryo electroporated with pCIG-Annexin A6 (green) after 6 (A), 5 (B,C), and 4 (D) hours of incubation and processing by immunohistochemistry for Claudin-1 (A), Cingulin (B), Cadherin6B (C), and N-cadherin (D) (all red), respectively. Arrows denote the loss of proteins on the electroporated side (right) of the neural tube. Scale bar in (A) is 50 µm and applicable to all images. GFP, green; DAPI, blue.

To ascertain whether Annexin A6 overexpression increased neural crest cell emigration by impacting EMT, we again examined molecular markers of adherens and tight junctions (Fig. 9). Although we observe no alterations in Claudin-1 and Cingulin localization (Fig. 9A,B; 4/4 embryos for each), we detect the premature downregulation of both Cadherin6B and N-cadherin (Fig. 9C,D, arrows; 5/5 and 6/6 embryos for Cadherin6B and N-cadherin, respectively) on the sides of embryos electroporated with pCIG-Annexin A6, with no change noted in the pCIG controls (data not shown). These results lend further credence to the hypothesis that Annexin A6, directly or indirectly, regulates neural crest cell EMT and emigration. Taken together, these overexpression data point to a phenotype of enhanced neural crest cell emigration and migration upon overexpression of Annexin A6 that can be associated with an expansion of the premigratory neural crest cell domain and alterations in the molecular changes underlying EMT. Importantly, our collective results have uncovered a role for Annexin A6 in controlling cranial neural crest cell emigration and migration.

## Discussion

Annexins constitute a large family of proteins that can reversibly associate with the plasma membrane. Originally discovered in the chicken growth plate cartilage [7], Annexin A6 has now been found to mediate a variety of functions due to its cytosolic and membrane localization, including the regulation of cell adhesion and motility through an interaction with the actin cytoskeleton [3]. Given these functions for Annexin A6, we reasoned it would be a good candidate to regulate neural crest cell ontogeny and thus we sought to define its role in the developing embryo.

*Annexin A6* transcripts are observed as early as the 4 ss in the neural tube at both cranial and trunk axial levels, and migratory cranial neural crest cells also express *Annexin A6*. Although we have not yet delineated the role of Annexin A6 in trunk neural crest cell emigration, it will be intriguing to see if Annexin A6 function in the head is also conserved at this axial level. Due to the lack of an effective commercially available antibody, we were unable to document Annexin A6 protein distribution in the developing embryo by immunohistochemistry; however, we can detect Annexin A6 protein in the neural tube by immunoblotting. Our results reveal the presence of *Annexin A6* in the developing midbrain neural crest cell population and indicate that Annexin A6 could play a potential role in neural crest cell development.

### Depletion of Annexin A6 Reduces the Size of the Premigratory and Migratory Neural Crest Cell Domains and Disrupts Neural Crest Cell EMT

To better delineate a functional role for Annexin A6 during neural crest cell ontogeny, we depleted Annexin A6 from the developing midbrain neural crest cell population using an *Annexin A6* MO. Knock-down of Annexin A6 reduces the size of the premigratory neural crest cell population, as noted by diminished *Snail2* expression in premigratory neural crest cells within the embryonic dorsal neural tube. This decrease is associated with a subsequent reduction in the migratory neural crest cell domain at various times post-MO electroporation. This effect on migration is apparent upon examination of a wide variety of neural crest cell molecular markers, including *Snail2*, *Sox10* and HNK-1. Furthermore, the change in the size of the migratory neural crest cell domain is not due to any alterations in cell size/shape or to changes in cell proliferation or cell death within the neuroepithelium or migratory neural crest cell population. In addition, adherens junction proteins are maintained upon depletion of Annexin A6, thereby ascribing a direct or indirect role for Annexin A6 in modulating the EMT process itself. Therefore, Annexin A6 regulates neural crest cell emigration and migration by impacting the premigratory neural crest cell population and their subsequent EMT and exit from the dorsal neural tube.

### Annexin A6 Overexpression Augments the Size of the Premigratory and Migratory Neural Crest Cell Domains and Enhances Neural Crest Cell EMT

To corroborate our MO knock-down data, we performed Annexin A6 overexpression experiments in the chick midbrain. We find that elevated levels of Annexin A6 expand the migratory neural crest cell domain, as assessed by whole-mount *in situ* hybridization for *Snail2* and *Sox10* and as well as immunohistochemistry for HNK-1. We often times observe cells in the neural tube lumen that are positive or negative for neural crest cell molecular markers. These results suggest that delaminated neural crest cells move in an inappropriate direction into the neural tube lumen. Alternatively, those cells negative for neural crest markers may in fact be more ventrolateral neuroepithelial cells that have been forced out of the neural tube upon Annexin A6 overexpression. We see this enhanced emigration phenotype at several time points post-electroporation, lending further credence to our results. In addition, we do not observe any appreciable differences in cell proliferation or cell death upon Annexin A6 overexpression. An expansion in the premigratory neural crest cell domain provides a correlative molecular explanation for this increased migratory neural crest cell population, evaluated by performing whole-mount *in situ* hybridization in young embryos prior to neural fold fusion for the premigratory neural crest cell marker *Snail2*. Finally, Annexin A6 directly or indirectly regulates the neural crest cell EMT program as noted by precocious loss of adherens junction proteins upon Annexin A6 overexpression. These data corroborate our knock-down data and further implicate Annexin A6 in the modulation of neural crest cell emigration.

Our results establish a novel and important role for an annexin family member in the formation of cranial neural crest cells during vertebrate development. Importantly, our data reveal correlations between changes in the size of the cranial premigratory and migratory neural crest cell populations upon perturbation of Annexin A6 and highlight a direct or indirect role for Annexin A6 in regulating neural crest cell EMT at the level of adherens junction disassembly. As such, Annexin A6 levels must be tightly controlled during embryogenesis in order to permit the appropriate development of premigratory neural crest cells and their subsequent EMT and emigration from the dorsal neural tube. Taken together, our work will provide the foundation for future studies aimed at elucidating the importance of Annexin A6 localization in neural crest cell differentiation and delineating the potential role of other annexin family members in the neural crest.

## Materials and Methods

### Chicken Embryo Culture

Fertilized chicken eggs were obtained from Hy-Line North America, L.L.C. (Elizabethtown, PA) or B & E Farms (York, PA) and incubated at 38°C in humidified incubators (EggCartons.com, Manchaug, MA). Embryos were staged according to the number of pairs of somites.

### Design and Electroporation of *Annexin A6* Antisense Morpholino

A 3′ lissamine-labeled antisense *Annexin A6* morpholino (MO),

5′-TCCTTTGGGTGCCATGGGAATCGC-3′, was designed to target the *Annexin A6* mRNA according to the manufacturer’s criteria (GeneTools, L.L.C.). A 5 bp mismatch lissamine-labeled antisense *Annexin A6* control MO.

5′-TCgTTTcGGTcCCATGcGAATCcC-3′ (mutated bases are in lower case; GeneTools, L.L.C.) was used that does not target *Annexin A6* mRNA. MOs were introduced into the developing chick embryo using a modified version of the electroporation technique [18]. Briefly, MOs were injected at a final concentration of 500 µM [19], [20] into the neural tube lumen at the desired axial level and 2, 25 volt, 30 mSec pulses were applied across the embryo.

### Overexpression of Annexin A6 *in vivo*

The full-length *Annexin A6* cDNA was directionally cloned into the pCIG chick expression construct by PCR using a chick cDNA library (7–12 ss) as the template in order to produce pCIG-Annexin A6 and sequenced to confirm accuracy. The control (pCIG) or pCIG-Annexin A6 expression construct was introduced into the embryo at a concentration of 3 µg/µl, as described above for the MO electroporations.

### Whole-mount *in situ* Hybridization

Whole-mount *in situ* hybridization was performed as described previously in [19], [20], [23]. Stained embryos were viewed in whole-mount at room temperature in 70% glycerol using a Zeiss SteREO Discovery.V8 compound fluorescent microscope. Images were captured using the Zeiss Axiovision Rel 4.6 software with the Zeiss Axiocam MRc5 camera. Transverse sections were obtained by cryostat-sectioning gelatin-embedded embryos at 14 µm in a Leica Frigocut or Fisher Microm cryostat, and coverslips were mounted on processed sections using Fluoromount G (Fisher). Sections were viewed at room temperature using a Zeiss AxioObserver.Z1 inverted microscope, and images were acquired using the Zeiss Axiovision Rel 4.6 software with the Zeiss Axiocam HRC camera. All exported images were processed in Adobe Photoshop 9.0 (Adobe Systems).

### Immunohistochemistry

Immunohistochemical detection of various proteins was performed in whole-mount or on transverse sections following 4% paraformaldehyde fixation of embryos and cryostat-sectioning, as in [19], [20]. The following primary antibodies and concentrations were used in the experiments: phospho-histone H3 (Millipore Ser10; 1∶500); GFP (Invitrogen A11122 or A11121; 1∶500); and HNK-1 (1∶100). Immunostaining for Cadherin6B, N-cadherin, Cingulin, and Claudin-1 and TUNEL assays were performed as described in [19], [20]. Appropriate fluorescently-conjugated secondary antibodies (Alexa 488 or 594) from Invitrogen were used at a concentration of 1∶200 to 1∶1000. Imaging of sections and data processing were carried out as described above for *in situ* hybridizations using the Zeiss AxioObserver.Z1 inverted microscope and Adobe Photoshop 9.0 (Adobe Systems), respectively. Sections were stained with DAPI to mark cell nuclei and mounted using Fluoromount G (Fisher).

### Immunoblotting

Midbrain neural tube halves electroporated with expression constructs or MOs and re-incubated for 8 hours, along with the contralateral control side halves, were dissected out of the embryo using tungsten needles. Neural tube halves were pooled and flash-frozen in liquid nitrogen. Tissue was then resuspended in Lysis Buffer (50 mM Tris, pH8, 150 mM NaCl, 1% NP-40) supplemented with a protease inhibitor cocktail (Roche) and 0.1M PMSF. Whole-cell lysates were prepared by incubating tissue with Lysis Buffer on ice for 20 minutes as in [21]. The soluble fraction was collected by centrifugation and protein concentration was determined using a Bradford assay (Biorad). Equivalent amounts of protein (50 µg) were boiled in 2X SDS sample buffer, loaded and separated by SDS-PAGE (10% gel), and then transferred to 0.45 µm PVDF membrane (BioTrace). Membranes were incubated in 5% skim milk in PTW (1× PBS +0.1% Tween-20; Blocking solution) for 30 minutes at room temperature and then incubated overnight at 4°C with the following primary antibodies at a 1∶1000 dilution in the Blocking solution: Anti-Annexin VI (Abcam, ab31026); β-actin (Santa Cruz, C4, sc-47778). Membranes were washed in PTW and then incubated with secondary antibodies conjugated to HRP (Annexin A6: Rockland, goat anti-rabbit IgG, #611-1302, 1∶30,000; β-actin: Jackson Immuno Research, goat anti-mouse IgG_1_, #115-035-205, 1∶10,000) in Blocking solution for 1 hour at room temperature. Protein detection was performed using an equal volume (1∶1) of ECL reagents (Super West Pico or Femto substrates, ThermoScientific) for 5 minutes and visualized and quantified using a ChemiDoc XRS system (Biorad). Fold differences in Annexin A6 protein levels were calculated after normalizing to the loading control (β-actin) and comparing to Annexin A6 levels 1) on the contralateral control side of the embryo and 2) neural tube halves electroporated with the control MO or pCIG construct.

### Cell Counts

Cell counts of *Sox10*- and *Snail2*-expressing cells in sections were performed as described previously [19], [20], [23]. Briefly, embryos electroporated with control MO, *AnnexinA6* MO, pCIG or pCIG-Annexin A6 and hybridized with a *Sox10* or *Snail2* antisense riboprobe were imaged and subsequently cryostat-sectioned at 14 µm. Sections were stained with DAPI to enable the identification of individual nuclei and mounted for imaging using Fluoromount G (Fisher). 7–10 serial section images from the midbrain were captured in a minimum of 3 embryos that had MO or GFP localized to the dorsal neural tube (and thus successfully electroporated). All DAPI-positive nuclei surrounded by cytoplasmic *Sox10* in the migratory neural crest cell streams (or *Snail2* in premigratory neural crest cells in the dorsal neural tube) on both the electroporated and contralateral control side were counted and recorded. Fold differences were then averaged over the number of sections in which cells were counted, and the standard error of the mean was calculated and compared for embryos electroporated with either control MO, *AnnexinA6* MO, pCIG or pCIG-AnnexinA6. Significance of results was established using the unpaired Student’s *t* test.

## References

[1] Kalcheim C, Le Douarin N (1999) The neural crest. New York: Cambridge University Press.

[2] Gerke V, Moss SE (2002) Annexins: from structure to function. Physiol Rev 82: 331–371. 11917092 10.1152/physrev.00030.2001

[3] Grewal T, Koese M, Rentero C, Enrich C (2010) Annexin A6-regulator of the EGFR/Ras signalling pathway and cholesterol homeostasis. Int J Biochem Cell Biol 42: 580–584. 20044025 10.1016/j.biocel.2009.12.020

[4] Gerke V, Creutz CE, Moss SE (2005) Annexins: linking Ca2+ signalling to membrane dynamics. Nat Rev Mol Cell Biol 6: 449–461. 15928709 10.1038/nrm1661

[5] Avila-Sakar AJ, Creutz CE, Kretsinger RH (1998) Crystal structure of bovine annexin VI in a calcium-bound state. Biochim Biophys Acta 1387: 103–116. 9748523 10.1016/s0167-4838(98)00111-3

[6] Avila-Sakar AJ, Kretsinger RH, Creutz CE (2000) Membrane-bound 3D structures reveal the intrinsic flexibility of annexin VI. J Struct Biol 130: 54–62. 10806091 10.1006/jsbi.2000.4246

[7] Cao X, Genge BR, Wu LNY, Buzzi WR, Showman RM, et al (1993) Characterization, Cloning and Expression of the 67-kDa Annexin from Chicken Growth Plate Cartilage Matrix Vesicles. Biochem Biophys Res Commun 197: 556–561. 8267590 10.1006/bbrc.1993.2515

[8] Smythe E, Smith PD, Jacob SM, Theobald J, Moss SE (1994) Endocytosis occurs independently of annexin VI in human A431 cells. J Cell Biol 124: 301–306. 7905003 10.1083/jcb.124.3.301 PMC2119942

[9] Vila de Muga S, Timpson P, Cubells L, Evans R, Hayes TE, et al (2009) Annexin A6 inhibits Ras signalling in breast cancer cells. Oncogene 28: 363–377. 18850003 10.1038/onc.2008.386

[10] Creutz CE, Snyder SL (2005) Interactions of annexins with the mu subunits of the clathrin assembly proteins. Biochemistry 44: 13795–13806. 16229469 10.1021/bi051160w

[11] Cubells L, Vila de Muga S, Tebar F, Wood P, Evans R, et al (2007) Annexin A6-induced alterations in cholesterol transport and caveolin export from the Golgi complex. Traffic 8: 1568–1589. 17822395 10.1111/j.1600-0854.2007.00640.x PMC3003291

[12] Cubells L, de Muga SV, Tebar F, Bonventre JV, Balsinde J, et al (2008) Annexin A6-induced inhibition of cytoplasmic phospholipase A2 is linked to caveolin-1 export from the Golgi. J Biol Chem 283: 10174–10183. 18245088 10.1074/jbc.M706618200

[13] Babiychuk EB, Draeger A (2006) Biochemical characterization of detergent-resistant membranes: a systematic approach. Biochem J 397: 407–416. 16608442 10.1042/BJ20060056 PMC1533311

[14] Monastyrskaya K, Babiychuk EB, Hostettler A, Wood P, Grewal T, et al (2009) Plasma membrane-associated annexin A6 reduces Ca2+ entry by stabilizing the cortical actin cytoskeleton. J Biol Chem 284: 17227–17242. 19386597 10.1074/jbc.M109.004457 PMC2719360

[15] Grewal T, Enrich C (2009) Annexins–modulators of EGF receptor signalling and trafficking. Cell Signal 21: 847–858. 19385045 10.1016/j.cellsig.2009.01.031

[16] Grewal T, Enrich C (2006) Molecular mechanisms involved in Ras inactivation: the annexin A6-p120GAP complex. Bioessays 28: 1211–1220. 17120209 10.1002/bies.20503

[17] Sakwe AM, Koumangoye R, Guillory B, Ochieng J (2011) Annexin A6 contributes to the invasiveness of breast carcinoma cells by influencing the organization and localization of functional focal adhesions. Exp Cell Res 317: 823–837. 21185831 10.1016/j.yexcr.2010.12.008 PMC3049817

[18] Itasaki N, Bel-Vialar S, Krumlauf R (1999) 'Shocking' developments in chick embryology: electroporation and in ovo gene expression. Nat Cell Biol 1: E203–207. 10587659 10.1038/70231

[19] Jhingory S, Wu CY, Taneyhill LA (2010) Novel insight into the function and regulation of alphaN-catenin by Snail2 during chick neural crest cell migration. Dev Biol 344: 896–910. 20542025 10.1016/j.ydbio.2010.06.006 PMC2914159

[20] Wu CY, Jhingory S, Taneyhill LA (2011) The tight junction scaffolding protein cingulin regulates neural crest cell migration. Dev Dyn 240: 2309–2323. 21905165 10.1002/dvdy.22735 PMC3177993

[21] Taneyhill LA, Coles EG, Bronner-Fraser M (2007) Snail2 directly represses cadherin6B during epithelial-to-mesenchymal transitions of the neural crest. Development 134: 1481–1490. 17344227 10.1242/dev.02834 PMC2595139

[22] Coles EG, Taneyhill LA, Bronner-Fraser M (2007) A critical role for Cadherin6B in regulating avian neural crest emigration. Dev Biol 312: 533–544. 17991460 10.1016/j.ydbio.2007.09.056 PMC2266065

[23] Wilkinson DG (1992) Whole mount in situ hybridization of vertebrate embryos. In: Wilkinson DG, editor. In Situ Hybridization. Oxford: Oxford University Press. 75–83.

